# 1814. Retrospective Study of Skin and Soft Tissue Actinomycosis in an Urban Pediatric Academic Medical Center

**DOI:** 10.1093/ofid/ofad500.1643

**Published:** 2023-11-27

**Authors:** Salih Demirhan, Erika Orner, Wendy Szymczak, Philip J Lee, Margaret Aldrich

**Affiliations:** Children’s Hospital at Montefiore, Albert Einstein College of Medicine, Bronx, NY; Montefiore Medical Center, Bronx, New York; Montefiore Medical Center, Albert Einstein College of Medicine, Bronx, NY; Children's Hospital at Montefiore, Bronx, New York; Children's Hospital at Montefiore, Albert Einstein College of Medicine, Bronx, New York

## Abstract

**Background:**

*Actinomyces* species are known colonizers of human mucosal surfaces but are rarely associated with disease in immunocompetent hosts. Pediatrics studies are limited to case reports. This retrospective study aims to describe the characteristics of skin and soft tissue actinomycosis (SSTA) and the frequency and risk factors of recurrence in pediatric patients.

**Methods:**

We conducted this study of patients aged ≤21 years with *Actinomyces* growth in abscess cultures obtained under sterile conditions between January 2019 and December 2022 (Figure 1). Patient demographics were collected using the EMR, and all patients had at least a one-year follow-up period in which to ascertain recurrence.

**Figure:**
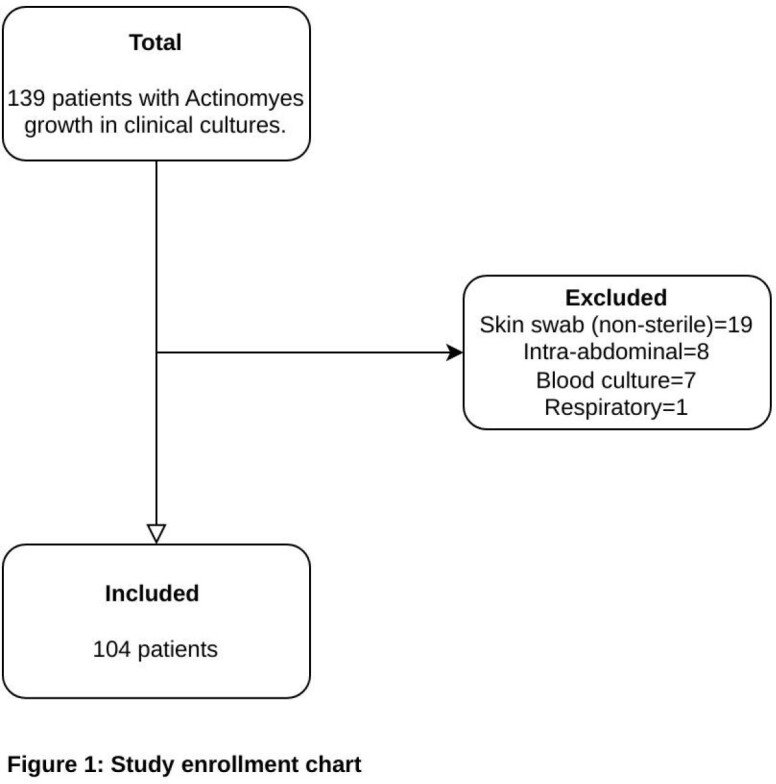

**Results:**

One hundred four patients met inclusion criteria; median age 19 (IQR 17-20) years, 68.3% female, 46.2% Black and 47.1% Hispanic. Body mass index (BMI) was > 25 in 59.8% of patients. *Actinomyces turicensis* (n=47) was the most commonly isolated subspecies and 71.2% of *Actinomyces* cultures were monobacterial. Clindamycin and trimethoprim-sulfamethoxazole were the most commonly used antibiotics. Only 7 patients had consultation with pediatric infectious diseases. Patients who had a prior abscess at the same anatomic site made up 29.8% of the study cohort and 33.7% had documented recurrence after the first SSTA episode, within median 10 (IQR 6-16) months after initial episode (Figure 2 and Table 1). Monobacterial culture growth (85.7% vs 63.8%, p=0.02), patients with BMI >25 (75% vs 52.6%, p=0.04) and patients with prior abscess in the same area (51.4% vs 18.8%, p=0001) were significantly higher in patients with recurrent actinomycosis (Table 2). In univariate analysis, monobacterial growth (OR= 3.4, 95% CI 1.2-9.9), BMI >25 (OR=2.7, 95% CI=1.1-7.0) and prior abscess (OR=4.6, 95% CI=1.9-11.2) were associated with increased odds of recurrence.

**Figure:**
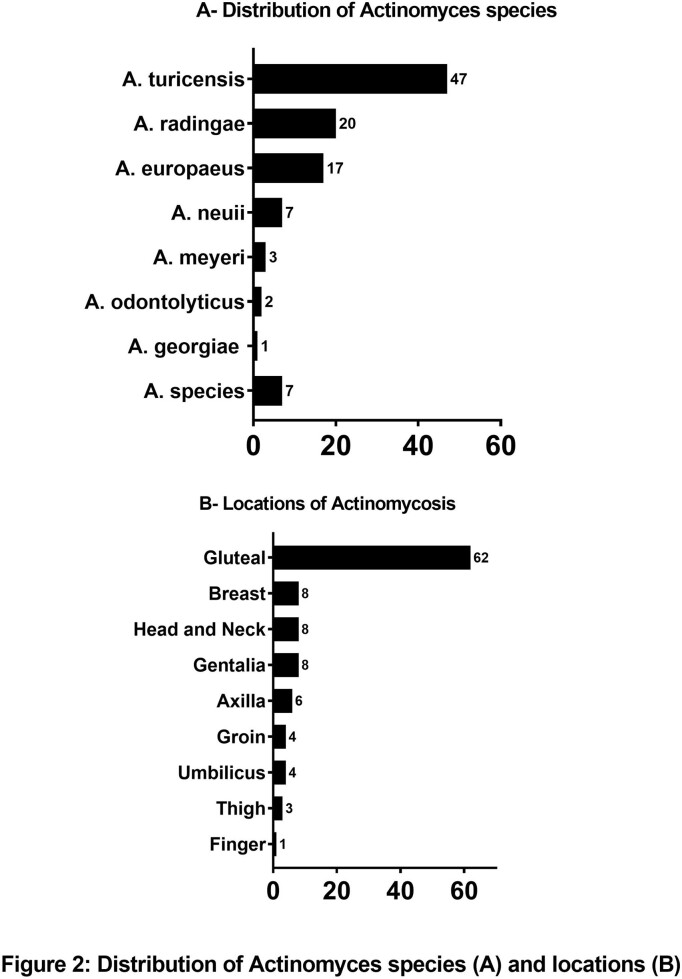

**Figure:**
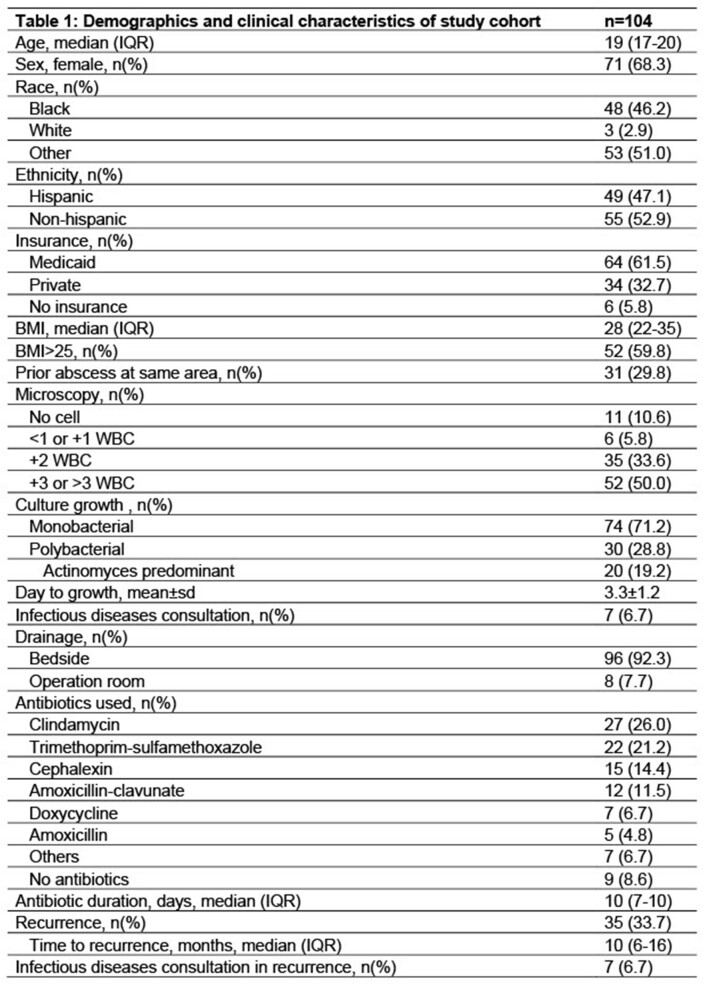

**Figure:**
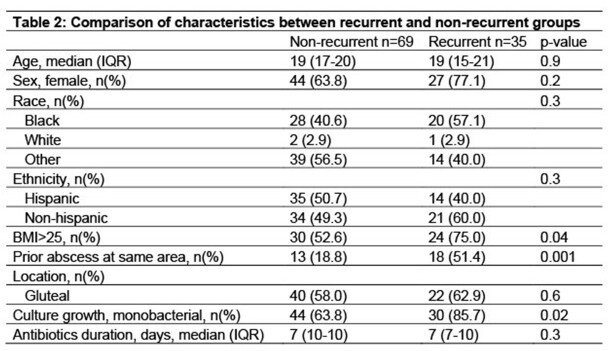

**Conclusion:**

Our findings suggest that patients with SSTA were generally adolescents with elevated BMI. Monobacterial culture growth, BMI >25, and prior abscess in the same area were significantly associated with recurrence. High recurrence rate, suboptimal antibiotic utilization and very low rates of consultation with infectious diseases suggest that providers should be informed and updated regarding this rare but difficult to treat infection.

**Disclosures:**

**All Authors**: No reported disclosures

